# Implantation of Adipose-Derived Mesenchymal Stromal Cells (ADSCs)-Lining Prosthetic Graft Promotes Vascular Regeneration in Monkeys and Pigs

**DOI:** 10.1007/s13770-023-00615-z

**Published:** 2024-01-08

**Authors:** Xiao Zuo, Pengfei Han, Ding Yuan, Ying Xiao, Yushi Huang, Rui Li, Xia Jiang, Li Feng, Yijun Li, Yaya Zhang, Ping Zhu, Hongge Wang, Ning Wang, Y. James Kang

**Affiliations:** 1 Regenerative Medicine Research Center, Sichuan University West China Hospital, Chengdu, 610093 China; 2 Sichuan 3D Bioprinting Institute, Chengdu, China; 3 Division of Vascular Surgery, Department of General Surgery, Sichuan University West China Hospital, Chengdu, China

**Keywords:** Adipose-derived mesenchymal stromal cells (ADSCs), 3D printing hybrid vascular graft, Long-term animal post-surgery, Vessel regeneration

## Abstract

**Background:**

Current replacement procedures for stenosis or occluded arteries using prosthetic grafts have serious limitations in clinical applications, particularly, endothelialization of the luminal surface is a long-standing unresolved problem.

**Method:**

We produced a cell-based hybrid vascular graft using a bioink engulfing adipose-derived mesenchymal stromal cells (ADSCs) and a 3D bioprinting process lining the ADSCs on the luminal surface of GORE-Tex grafts. The hybrid graft was implanted as an interposition conduit to replace a 3-cm-long segment of the infrarenal abdominal aorta in Rhesus monkeys.

**Results:**

Complete endothelium layer and smooth muscle layer were fully developed within 21 days post-implantation, along with normalized collagen deposition and crosslinking in the regenerated vasculature in all monkeys. The regenerated blood vessels showed normal functionality for the longest observation of more than 1650 days. The same procedure was also conducted in miniature pigs for the interposition replacement of a 10-cm-long right iliac artery and showed the same long-term effective and safe outcome.

**Conclusion:**

This cell-based vascular graft is ready to undergo clinical trials for human patients.

**Supplementary Information:**

The online version contains supplementary material available at 10.1007/s13770-023-00615-z.

## Introduction

Replacement of stenosis or occluded arteries with bypass grafts remains an important clinical intervention in advanced vascular disease [1, 2]. This is often accomplished by the use of autologous veins/arteries or by prosthetic conduits composed of materials such as Dacron or expanded polytetrafluoroethylene (ePTFE). Extensive efforts have been devoted to developing prosthetic vascular grafts since the use of the patient’s own vasculature is often limited by disease conditions or unavailability of suitable vasculature [3, 4]. However, the common and unresolved issue of small-diameter (≤ 6 mm) prosthetic grafts is the low patency caused by thrombosis and restenosis due to the lack of their biological structural and functional compatibility with native blood vessels [5]. It is thus needed to improve the prosthetic products for long-term patency in clinical application.

Tissue-engineered blood vessels (TEBV) have been extensively studied to address the above issue. A TEBV is composed of natural or synthetic biodegradable vascular scaffolds along with cells seeded on the surface of the scaffolds or simply cell-free scaffolds that can attract cells to grow on the surface *in vivo* [6]. TEBVs are designed to grow, remodel, and repair *in vivo* and eventually form blood vessels identical to native conduits [6, 7]. The strategy of TEBVs mainly relies on the coupling and integration between the scaffold and the seeded or recruited cells [7, 8] to provide safe and effective products for clinical applications. The complexity for designing and the difficulty for fabricating a clinically feasible TEBV are far beyond current research endeavors [9–12]. In particular, the prolonged production time makes TEBV unsuitable for on-demand clinical circumstances [13].

To address these issues, we explored the possibility of uncoupling the mechanical property from the cellularity process, providing immediate mechanical support and allowing the body to proceed with its own vascular regeneration process. We proposed a fabrication strategy of a hybrid TEBV. An ePTFE artificial vessel was selected as the conduit structure for mechanical support, and a uniform layer of adipose-derived mesenchymal stromal cells (ADSCs) [14] was “printed” on the luminal surface using a 3D bioprinting apparatus applying a bioink composed of ADSC biosynspheres. The 3D bioprinted ADSC vascular grafts were fabricated within two hours and immediately implanted for interposition replacement of the abdominal aorta in Rhesus monkeys and the iliac artery in pigs. We observed successful regeneration of the blood vessel in the ePTFE lumen, performing physiological vasculature function for more than four years.

## Materials and methods

### Animals

Male naive Rhesus (Macaca mulatta) monkeys (four to eight years old and six to nine kg, Sichuan Green-house Biotech) and naive Wuzhishan Inbred’ Miniature Pig (WZSP) (10 to 24 months old, 35 to 50 kg for males and 30 to 45 kg for females, Grand Life Science & Technology Ltd) were acclimated to the laboratory conditions for 21 days at the animal facility of Sichuan Huashen Veterinary Biological Products Co., Ltd.

The animals were treated in strict accordance with good animal practice under the supervision of veterinarians. Monkeys were housed in individual stainless steel cages in a controlled environment under a 12-h light–dark cycle (lights on at 8:00 and off at 20:00), fed completely formulated monkey feed (twice per day, 100–150 g each time), and had free access to drinking water produced by a reverse osmosis system. In addition, seasonal fresh fruits were provided three times weekly.

Environment enrichment includes a metal mirror attached to each cage, toy playing (plastic balls and swing ring) and video watching twice a week. Pigs were group-housed in the pigpen during the acclimation period, and postgraft implantation animals were housed individually in stainless steel cages in a facility. The facility had a permit for laboratory animal use. The animals were fed twice daily with certified experimental minipig maintenance feed supplied by Beijing Keao Xieli Feed Co., Ltd.

The diet is routinely analyzed by the manufacturer for nutritional components and environmental contamination. Qualified drinking water was supplied via an automatic drinking tap or water bowl ad libitum. Water is routinely analyzed for contaminants and specific microbes, and available information indicates that no contaminants present in the drinking water at a concentration likely influence the outcome of this study. All animal procedures described here were approved by the Institutional Animal Care and Use Committee at Sichuan University West China Hospital.

### Adipose-derived mesenchymal stromal cells (ADSCs)

ADSCs were isolated from rhesus monkeys or miniature pigs as follows: Approximately five g (1 mm^3^) adipose tissue was harvested from the lateral hypogastric region (monkey) or the chest region (swine) after sterilization from anesthetized animals by aseptic surgery. ADSCs were isolated following a procedure described previously [15, 16]. The isolated ADSCs were propagated for three generations within 14 days, and the propagated ADSCs were cryopreserved until the use of biosynsphere bioinks.

### Bioink preparation and 3D bioprinting

Cell-laden microgels were prepared according to a Biosynsphere^®^ (Revotek Co., Ltd) technology [17] and used to prepare the stromal cell-based bioink for the fabrication of the ADSC vascular grafts. The bioink was a mixture of sterilized collagen Type I (17 mg/mL) and biosynspheres in a volumetric ratio of 2:1. An in-house 3D bioprinter was used to “print” the bioink into the lumen structure, and then the bioink was adhered to the surface of the endovascular lumen of ePTFE prosthetic grafts by tissue adhesive. The parameters of 3D bioprinter are described in the Table 1. The ePTFE prosthetic grafts used in this study were obtained from W.L. Gore & Associates, Inc. The main component of tissue adhesive is α-N-octyl cyanoacrylate, and the tissue adhesive was obtained from Guangzhou Baiyun Medical Glue Co. LTD, China.

**Table 1:** The parameters of in house 3D bioprinter for the construction of the 3D bioprinted ADSC vascular graft

Parameters	Value
Printing temperature	37 °C
Nozzle volumetric speed	10 μL/s
Nozzle movement velocity	54,000 mm/min
Cell number/cm	8 × 10^6^

### Determination of the stemness of ADSCs

The surface marker of ADSCs was analyzed by flow cytometry with the following antibodies used: (1) CD90/CD44-PE (BD Pharmingen, US, 561970, 561858), (2) CD73/HLA-DR-PerCP-CyTM5.5 (BD Pharmingen, US, 561260, 552764), (3) CD29-APC (BD Pharmingen, US, 561794), (4) CD45/CD31-FITC (BD Pharmingen, US, 557803, 557508), and (5) CD105-PE-Cy7 (eBioscience, US, 25-1057). The induced differentiation of ADSCs was performed by culturing the ADSCs in conditioned induction medium (Cyagen, US). Alizarin red staining was used to detect the osteoblast differentiation of ADSCs. Oil red O staining was used to detect the adipogenic differentiation of ADSCs. Alcian blue staining was used to detect the chondrogenic differentiation of ADSCs.

### Vascular graft implantation

A total of 30 male Rhesus monkeys four to eight years old (approximately six to nine kg body weight) were subjected to routine aseptic surgical procedures as described previously [18–20]. A 3-cm-long segment of the infrarenal abdominal aorta was extracted through a midline laparotomy incision. The bioprinted ADSC vascular graft was implanted by End-to-end anastomosis to replace the 3-cm-long segment. Anticoagulant (heparin, i.v., 0.5 mg kg^−1^) was given immediately after the operation and every day thereafter for the first and maximum five days post-operation.

They were given an anticoagulant, low molecular weight heparin (LMWH), only for the first 5 days (unless sampling less than five days) immediately post-implantation. Histological and immunohistochemical analyses were performed on the graft samples harvested after implantation at 4 and 24 h and on 5, 7, 14, 21, 28, and 70 days. There were three monkeys allowed to survive for an indefinite time after implantation and subjected to long-term noninvasive monitoring for blood vessel function unless otherwise specified.

A total of 34 miniature pigs 10 to 24 months old (approximately 35–50 kg body weight for males, approximately 30–45 kg for females) were subjected to routine aseptic surgical procedures and three pigs remained untreated as controls. The segment of the infrarenal abdominal aorta and left external iliac aorta were exposed and extracted through a midline laparotomy incision. Approximately 10 cm long 3D bioprinted ADSC vascular grafts (26 pigs) or ePTFE grafts (eight pigs) were bypassed from the infrarenal abdominal aorta to the left external iliac artery. The 3D bioprinted ADSC vascular grafts or ePTFE grafts were anastomosed end-to-side to the infrarenal aorta with 6-0 Prolene sutures, and the 3D bioprinted ADSC vascular grafts or ePTFE grafts were anastomosed end-to-side to the left iliac artery with 7-0 Prolene sutures. The starting section of the left iliac artery was ligated to simulate the clinical setting of artery occlusion, and the left iliac artery was replaced by 3D bioprinted ADSC vascular grafts or ePTFE grafts.

### Immunohistochemistry/immunofluorescence staining

The vascular grafts or regenerated vessels explanted from monkeys and pigs were processed for histological and immunohistochemical analyses as described previously (21). The following antibodies were used: rabbit anti-Oct4 polyclonal antibody (Abcam, US, ab19857), mouse anti-CD31 monoclonal antibody (Maixin, China, MAB-0031), goat anti-CD31 (R&D, US, AF3628), rabbit anti-α-SMA polyclonal antibody (Abcam, US, ab5694), rabbit anti-calponin monoclonal antibody (Abcam, US, ab46794), rabbit anti-SMM-HC (Myh11) monoclonal antibody (Abcam, US, ab133567), rabbit anti-collagen I polyclonal antibody (Abcam, US, ab34710), rabbit anti-PGP9.5 monoclonal antibody (Abcam, US, ab108986), donkey anti-goat IgG (H+L) cross-adsorbed secondary antibody, Alexa Fluor 488 (Thermo Fisher, US, A11055), goat anti-rabbit IgG (H+L) cross-adsorbed secondary antibody, Alexa Fluor 568 (Thermo Fisher, US, A11011), and SignalStain Boost IHC Detection Reagent (HRP, Rabbit/Mouse) (Cell Signaling Tech., Tech., US, 8114/8125). All the tissues prepared for staining were harvested from the middle part of the implants.

### Ultrasound examination

For monkeys, after fasting for eight hours (water provided ad libitum), sedation was induced by intramuscular injection of ketamine (10 mg kg^−1^) and midazolam (1 mg kg^−1^). Monkeys were subjected to ultrasound evaluation with a 3.5-MHz ultrasonic probe (L9-3, iU22, Philips Medical Systems) in the supine position. B-mode, color Doppler and pulsed Doppler ultrasound were performed to examine abdominal aorta hemodynamics. Longitudinal and transverse views of the abdominal aorta (AO) were examined.

For pigs, the animal was sedated by intramuscular injection of Zoletil^®^ 50 (50 mg/mL) with 3–3.5 mL (approximately three to five mg/kg). Color Doppler ultrasound examination was performed on the engraftment area (ePTFE or 3D bioprinted ADSC vascular grafts) or sham area to evaluate blood flow through the implanted vessel graft, including vascular stenosis or occlusion and thrombosis detection, at or near the graft section.

### Computed tomography angiography (CTA) examination

For monkeys, before the test, sedation was induced by intramuscular injection of ketamine (10 mg·kg^−1^) and midazolam (one mg·kg^−1^), and then vein catheter access was established through a 22-gauge venous indwelling needle via the small vein on the right forearm for contrast delivery. X-ray images of the abdominal aorta were captured using a CT scanner (Lightspeed, GE). An iodine-containing contrast dye was injected intravenously to improve the quality of the images. Then, axial images were reconstructed and used to generate high-quality multiplanar reformatted images.

For pigs, the animal was sedated by intramuscular injection of Zoletil^®^ 50 (50 mg/mL) with 3–3.5 mL (approximately three to five mg/kg). In addition, before the CTA test, IV-line access was established through a 20-gauge venous indwelling needle via the marginal ear vessels for CTA contrast agent delivery. CTA examination was conducted on the engraftment area using intravenous injection of iodine-rich contrast agent (iopamidol) to evaluate blood flow through the implanted vessel graft, detect malformation of the implanted graft, and identify abnormalities of vascular stenosis or occlusion and thrombosis at or near the graft section.

### Statistical analysis

Data were obtained from three separate experiments and expressed as the means ± standard errors of the means (SEM). A single factor design was applied to this study. After a significant interaction was detected by analysis of variance (SPSS), the significance of the main effects was further determined by T test. The level of significance was considered when *P* < 0.05.

## Results

### Fabrication of the 3D bioprinted ADSC vascular graft and assembly of the hybrid TEBV

**Fig. 1:**
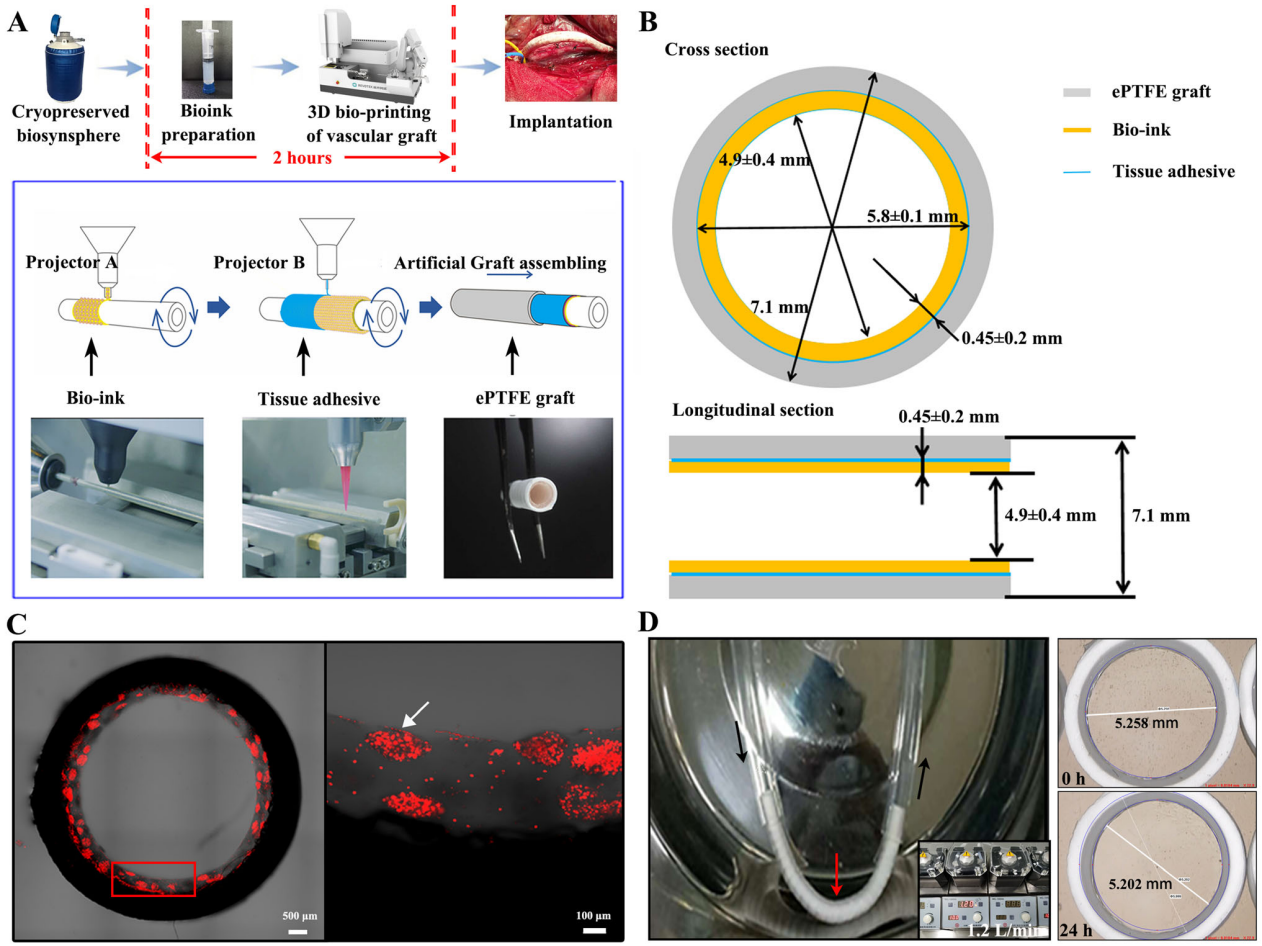
Fabrication and characterization of the 3D bioprinted ADSC vascular grafts. **A** Schematic diagram of the 3D bioprinted ADSC vascular graft preparation process. **B** Dimensional illustration of the 3D bioprinted ADSC vascular grafts. **C** Images of cell-laden biosynspheres in the 3D bioprinted ADSC vascular grafts. Cells were stained by red dye and cell clusters as one indicated by the white arrow were biosynspheres. **D** Integrity validation of the 3D bioprinted ADSC vascular grafts before and after 1.2 L/min of plasma substitute flushing for 24 h. Black arrows indicate the flow direction and the red arrow indicate the 3D bioprinted ADSC vascular graft

Autologous ADSCs freshly isolated from fat tissue were propagated in cultures avoiding any induction of differentiation (Fig. S1 in Supplementary File). The expanded ADSCs were encapsulated via a Biosynsphere^®^ technology as cell-laden microspheres for cryopreservation and cell protection [17]. On-demand preparation of the 3D bioprinted ADSC vascular graft was performed about two hours before implantation. The biosynspheres were thawed and mixed evenly with collagen type I to form the bioink for better mechanical properties. The hybrid TEBV was assembled in two consecutive steps: fabrication of a 3D bioprinted ADSC lining graft followed by its adherence to the luminal surface of the ePTFE prosthetic graft (Fig. 1A).

The bioink was applied to an in-house printer to be extruded on an air bladder enveloped rotary rod and warmed for 30 min to form a hollow tubular gelation. After that, a tissue adhesive was spread on the surface so that when an ePTFE graft was placed concentrically to the rod, the air bladder was inflated to cast and glue the bio-ink to the luminal surface of the ePTFE graft to form the 3D bioprinted ADSC vascular graft (Fig. 1A, and Movie S1 in Supplementary File).

The hybrid TEBV was fabricated with an outer diameter of 7.1 mm (as the diameter of the ePTFE graft) and an inner diameter of 4.9 ± 0.4 mm, and the thickness of the bioink layer was approximately 0.45 ± 0.2 mm (Fig. 1B). The cell-laden biosynspheres were dispersed in the bioink layer which closely adhered to the inner surface of the ePTFE graft, generating a smooth luminal surface (Fig. 1C). There was no significant difference in the mechanical property between the hybrid TEBV and the ePTFE graft, including longitudinal tensile strength, probe burst strength, suture strength, circumferential tensile strength and strength after repeated puncture (Fig. S2 in Supplementary File). As illustrated in Fig. 1D, the bioink layer of the hybrid TEBV remained complete after the hybrid graft was flushed as a U-shape setup with plasma substitute for 24 h at a flow rate of 1.2 L/min. The flow rate was several times higher than the *in vivo* blood flow of vessels with a similar diameter. The inner diameter of the hybrid graft was slightly changed from 5.258 to 5.202 mm after flushing. There were no fragments or detached biosynspheres found in the flushing out liquid. This was in accordance with the rheology study of the bioink.

### Vasculature regeneration from implanted hybrid TEBV in monkeys

**Fig. 2:**
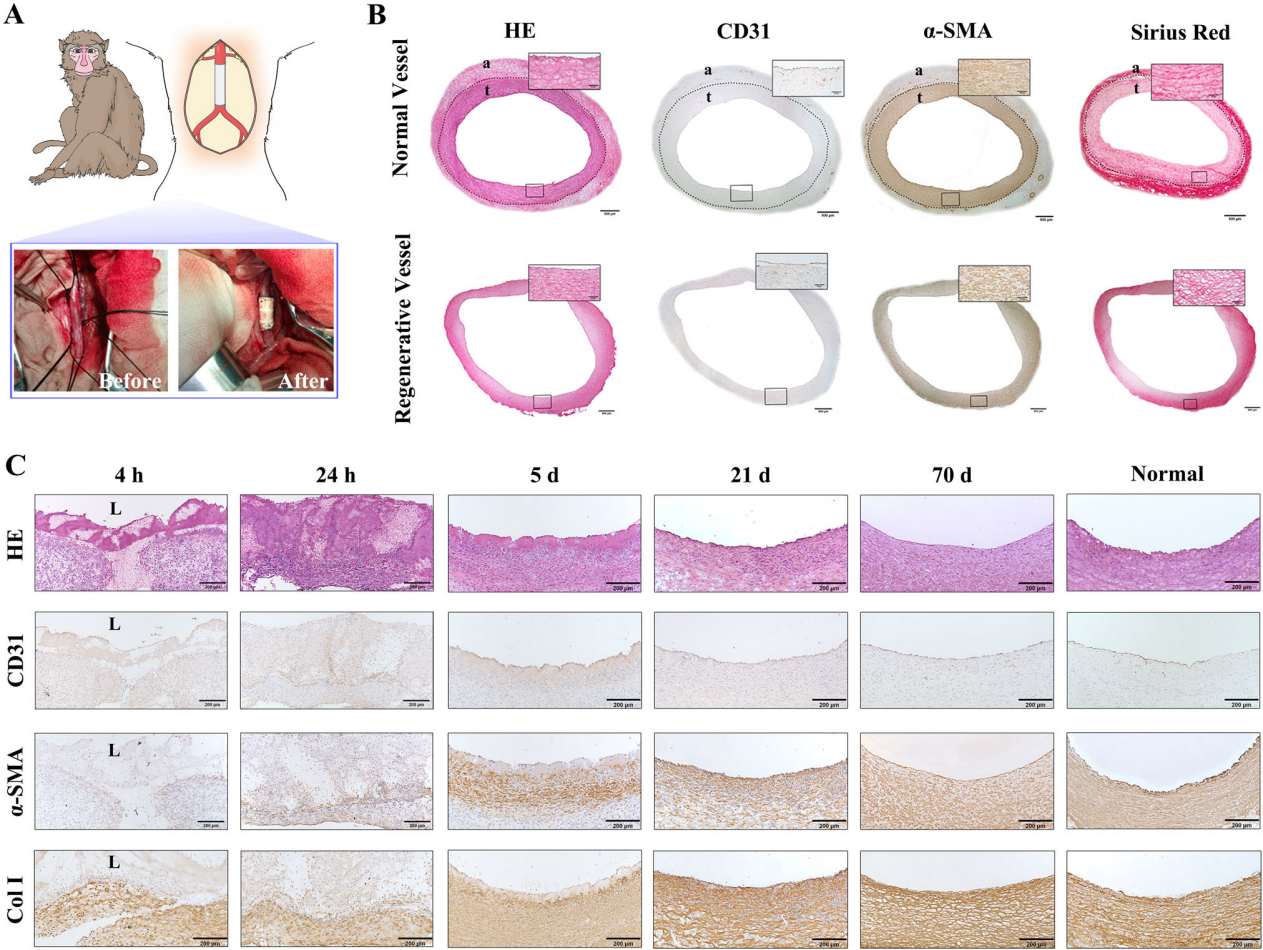
Histological and immunohistological analyses of the 3D bioprinted ADSC vascular grafts at different time points after implantation. **A** Schematic representation of 3D bioprinted ADSC vascular grafts implanted in a rhesus monkey. **B** Representative images of cross sections of normal abdominal aorta and the regenerative blood vessel. Cross sections were stained by hematoxylin/eosin (H/E) for overall structural observation, by CD31 for identification of endothelial cells, by α-smooth muscle actin (α-SMA) to define SMCs, or by Sirius red to detect total collagen fibers. The dashed line marks the boundary between the tunicae media vasorum and adventitia. a: adventitia. t: tunicae media vasorum. Bar = 500 μm in cross sections and bar = 50 μm in high power field. **C** Representative images of H/E staining and anti-CD31, anti-α-SMA, or anti-collagen I staining of the implanted vascular grafts at different time points after implantation. H&E staining shows the process of blood vessel tissue formation. Blood coagulation and inflammation were observed within 24 h after implantation. The dashed line shows the boundary between the printed vascular graft and the vessel lumen. Bar = 200 μm

Histological analysis of the 3D bioprinted ADSC vascular graft 70-day post-implantation in monkeys (Fig. 2A) revealed the formation of a complete and naturally identical endothelium as stained by human CD31 antibody. In addition, there was a naturally comparable smooth muscle layer regenerated. The analysis of collagen deposition and cross-linking, using Sirius Red staining of total collagens, identified normalized arrays of collagen fibers (Fig. 2B). However, there was no connective tissue in the outer layer of the newly regenerated vessel in comparison to the native vascular tissue. This would be affected by the ePTFE layer of the 3D bioprinted ADSC vascular grafts.

A time-course analysis revealed changes of the vascular graft started soon after the implantation (Fig. 2C). The infiltration of inflammatory cells was observed 24-h post-implantation. The endothelial layer started to appear on the 5th day post-implantation (Fig. 2C). A smooth muscle layer, as identified by human α-SMA antibody staining of smooth muscle cells (SMCs), was regenerated next to the endothelial layer. This smooth muscle layer also started to appear 5-day post-implantation and continued to evolve in subsequent days as the density of the SMCs increased over time. Collagen type I specific staining appeared at the 5th day post-implantation, and gradually normalized cross-linking of collagen type I was observed at the 21st day, and naturally comparable arrays of collagen type I fibers were formed at a later stage. Therefore, the naturally comparable vascular tissue layers, with the exception of the outer connective tissue layer, were regenerated 21-day post-implantation.

### Structural and functional normalization of the regenerated blood vessels

**Fig. 3:**
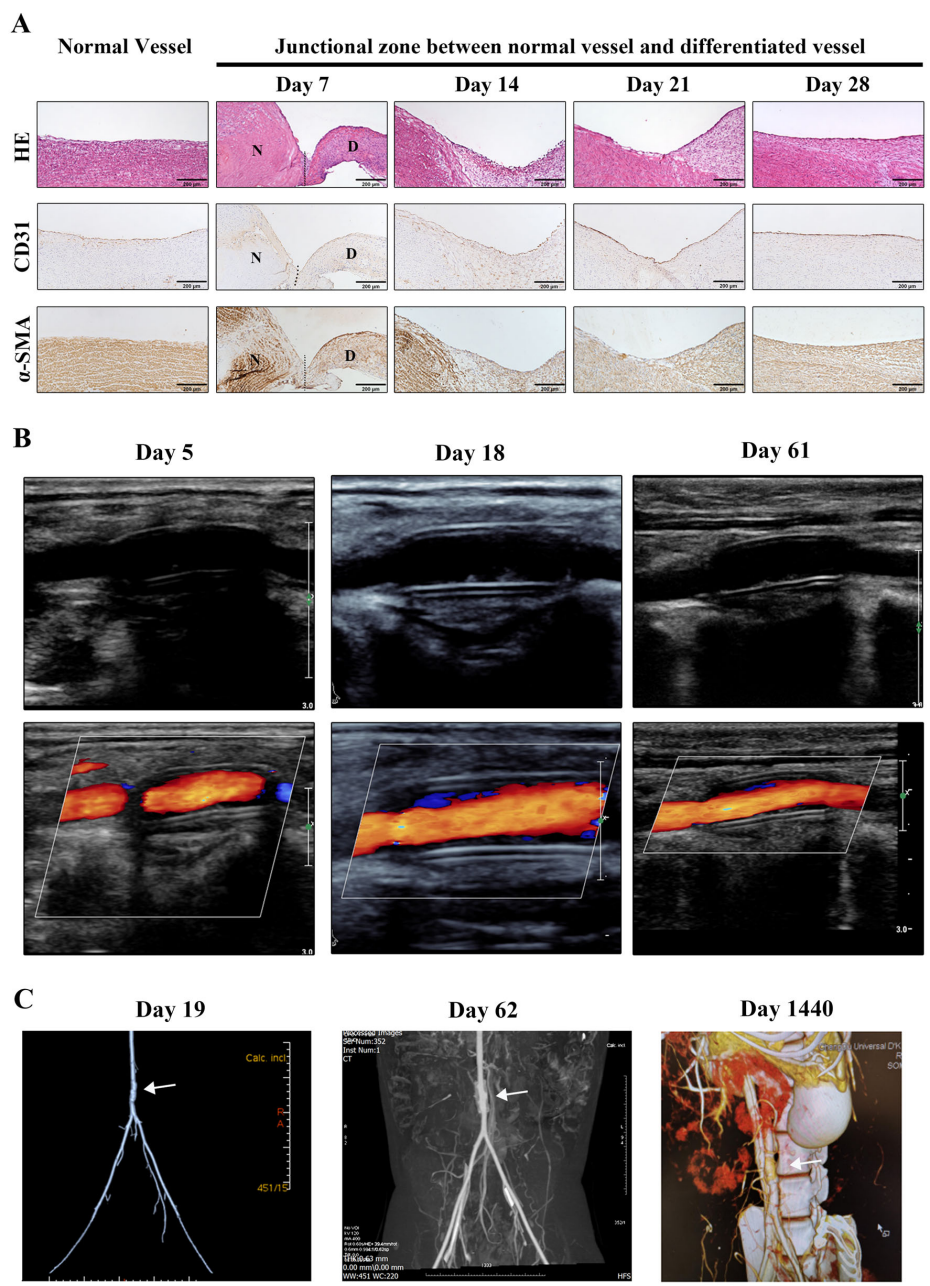
Histological and immunohistological analyses of the 3D bioprinted ADSC vascular graft at different time points after implantation. **A** Representative images of the junctional zone between the regenerated vessel and normal vessel at different time points. Fusion seemed to start on the 7th day and appeared to be complete on the 28th day after graft implantation. N: Normal vessel. D: 3D bioprinted ADSC vascular graft. Bar = 200 μm. The imaging for each time point was obtained from a separate individual, and more time points were obtained from other individuals showing the same changes. **B** High-resolution ultrasonography collected on the 5th, 18th, or 61st day after graft implantation shows an unobstructed lumen without thrombosis, stenosis or calcification. **C** Computed tomography angiography (CTA) performed on the 19th, 62nd or 1440th day after implantation shows sustained normality of the implanted ADSC vascular graft. The implanted ADSC vascular graft merged with normal vessels and departed from the ePTFE graft

The fusion between native vascular tissue and the implanted graft started 7-day post-implantation at the junction site. On the 28th day post-implantation, the juncture disappeared, and the regenerated vascular structure became indistinguishable from the native vascular tissue, as delineated by histological and immunohistological analysis (Fig. 3A).

A long-term observation of physiological function using iconography confirmed the normality of the blood vessel regenerated from the implanted 3D bioprinted ADSC vascular grafts. High-resolution ultrasonography results collected on the 5th, 18th, or 61st day showed that the lumen of the regenerated vessels was unobstructed, without thrombosis, stenosis or calcification (Fig. 3B). The regenerated blood vessel showed normal vascular pulsation (Movie S2 in Supplementary File), and the blood flow was uninterrupted (Movie S3 in Supplementary File). Computed tomography angiography (CTA) analysis performed on the 19th, 62nd and 1440th days after implantation showed a sustained normality of the regenerated blood vessel (Fig. 3C).

The ePTFE grafts covering the regenerated vascular structure in some monkeys were surgically removed on the 60th day after implantation. The blood vessel tissue obtained from a monkey at 1440-day post-implantation showed that the regenerated vessel remained structurally integrated, without any abnormality, forming a complete and continuous intima and middle membrane and outer membrane (Fig. S3A, B) in Supplementary File). In addition, nerves and small vessels were observed in the regenerated vascular tissue (Fig. S3C–E) in Supplementary File).

### Implantation of 3D bioprinted ADSC vascular grafts in miniature pigs

**Fig. 4:**
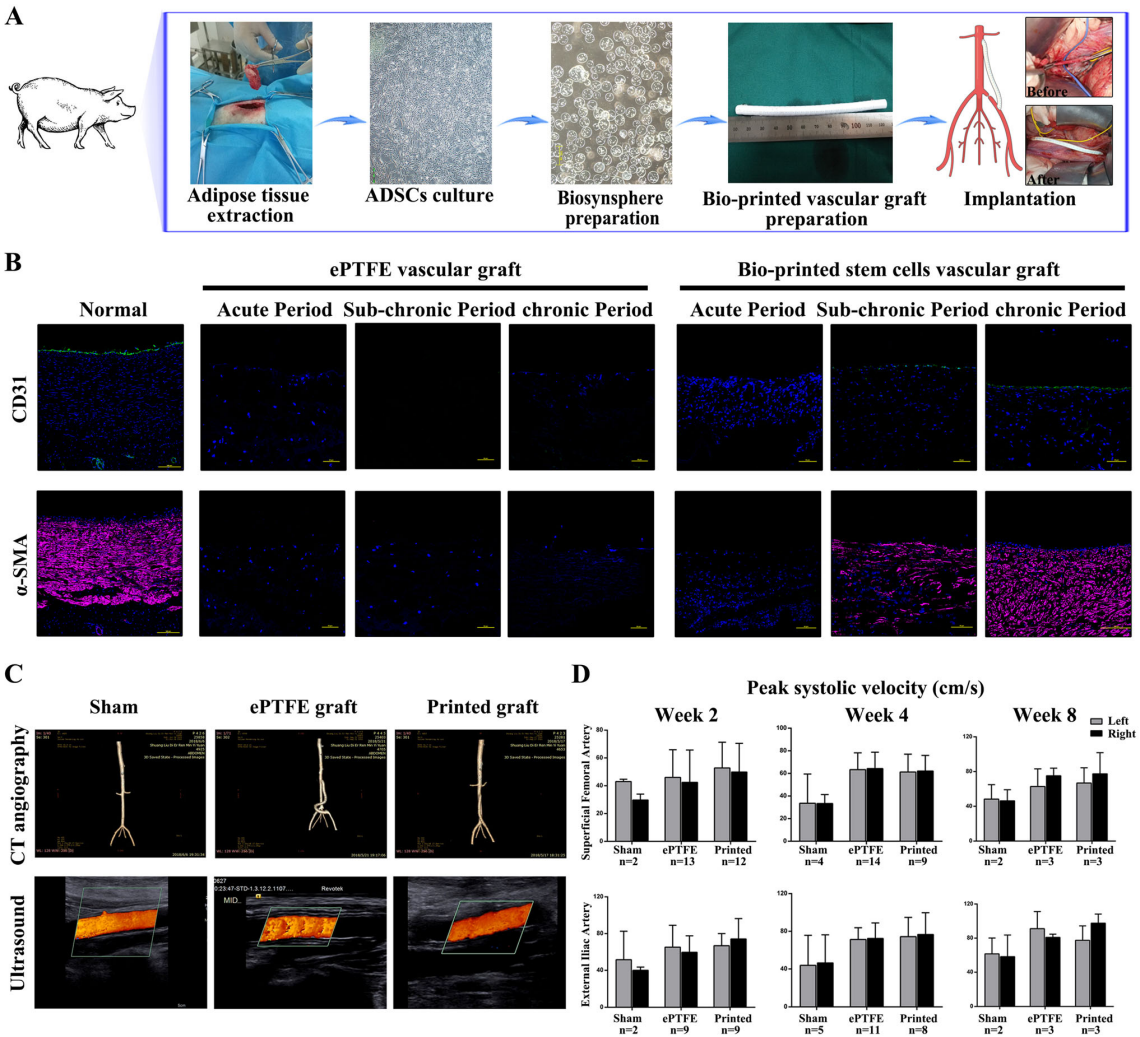
Histological, immunofluorescence and imagological analyses of bioprinted ADSC vascular grafts at different time points after implantation in Wuzhishan miniature pigs. **A** The protocol of the implantation experiment for ADSC hybrid grafts in Wuzhishan miniature pigs. **B** Representative images of immunofluorescence of the implanted ePTFE graft or ADSC-hybrid grafts at different time points after their implantation. Anti-CD31 staining and anti-α-SMA staining showed that both endothelial cells and SMCs began to form in the subchronic period after vascular implantation and gradually formed the complete endothelial cell layer and SMC layer with the extension of implantation time. **C** Computed tomography angiography (CTA) performed on the 62nd day after implantation shows sustained normality of the implanted ADSC cell vascular graft. High-resolution ultrasonography collected on the 62nd day after graft implantation showed an unobstructed lumen without thrombosis, stenosis or calcification. **D** There were no notable changes in peak systolic velocity among the sham group, ePTFE graft group and ADSC-hybrid graft group. ePTFE: ePTFE vascular graft. Printed: 3D bioprinted ADSC vascular graft

To further verify the clinical applicability of this strategy, we produced 3D bioprinted swine ADSC vascular grafts with a length of 10 cm and replaced them by interposition as a conduit from the abdominal aorta to the common iliac artery in miniature swine models (Fig. 4A). A total of 37 pigs underwent the same surgical implantation, and all of the animals survived. Another eight pigs were implanted with ePTFE grafts and three remained untreated as controls. The animals were sacrificed at the acute phase (3–5 days, n = 8), sub-chronic phase (21–28 days, n = 10) and chronic phase (> 45 days, n = 8).

Histopathological examination results showed that the implanted 3D bioprinted ADSC vascular grafts were gradually covered by an endothelial cell layer and SMC layer over time until the completion of tissue regeneration. There was no vascular regeneration found in the ePTFE graft control group (Fig. 4B). Imaging analysis showed that the implanted 3D bioprinted ADSC vascular grafts remained unobstructed, and there was no vascular stenosis (Fig. 4C). Hemodynamic tests showed that the blood flow in the implanted grafts was consistent with the contralateral normal vessels (Fig. 4D). A systemic test revealed that the implantation of the grafts did not affect the function of any organ system (Fig. S3 in Supplementary File).

## Discussion

Various strategies have been reported to construct TEBVs. However, the common unresolved issues for TEBV’s clinical translation are the unmatched material requirement for mechanical safety and cellularity, the synchronism between scaffold degradation and neo-tissue formation, and the long production time [9, 10, 13]. In this study, by uncoupling the mechanical property from the cellularity process, the strategy of the hybrid TEBV shows a great promise for overcoming the above issues. We used autologous ADSCs isolated from rhesus monkeys or miniature pigs in combination with the bio-inert and un-biodegradable ePTFE to successfully fabricate a hybrid TEBV. We generated naturally comparable and fully functional blood vessels from the bioprinted vascular graft. Regenerated endothelium could provide patients with a life-span solution for bypass replacement since the procedure outlined here can eliminate both the immune response and thrombosis for implanted prosthetic vascular grafts. The regeneration of functional endothelium and smooth muscle layers provides excellent support for patency, and the collagen deposition and normalized collagen fiber arrays in the regenerated vessels support a long-term conduit function. Our results proved that it is unnecessary for elaborately tuning the material properties of TEBVs. The solution and long-term results on large animals presented here could revolutionize the clinical procedures of arterial bypass.

The TEBV fabrication strategy presented in this study demonstrate a more practical potential of applying TEBV to clinical practice. In previous studies, complex biofabrication processes were employed to build TEBVs and days of culturing or maturation are necessary before implantation [21–23]. These procedures limit the on-demand use of TEBVs in clinical applications, especially in emergency treatments. In this study, the 3D bioprinted ADSC vascular graft can be fabricated within 2 h and used for implantation. To the best of our knowledge, this represents the shortest fabrication time comparing with previously reported TEBVs. Although autologous cells were applied in this study, it would work on allogeneic cells as well, since MSCs are known for their low immunogenicity as evidenced by even heterogenic MSC-based TEBV implantation [24, 25].

The use of mesenchymal stromal cells (MSCs), especially ADSCs have been reported in several studies involving TEBVs in recent years. There are studies using MSCs as cell sources for differentiation of adult cells to build TEBVs [22, 26]. However, the differentiation induction is time-consuming and has limitation in allogeneic transplantation. MSCs themselves are also utilized by their secretory, immunomodulatory, and fibrinolysis functions to help the regeneration of vascular tissue [27, 28]. The infiltration of inflammatory cells found at 24 h post implantation in our study was in accordance with previous reports as a sign of vascular regeneration beginning [29, 30].

There are limitations in the present study. The first is that the survival or engraftment of the ADSCs inside the body as well as the dosage of the cells have not been studied. Hashi et al*.* reported that MSCs can survival as long as seven days after implantation [31]. For clinical translation, these parameters are critical both for safety and effectiveness concerns. Moreover, a commercially available ePTFE prosthetic graft was chosen as the outer layer of the hybrid graft to lower the complexity of the study. ePTFE is a bio-inert material with no biodegradability which due to the presence of ePTFE on the surface of the implanted vascular graft there was a missing of the vascular adventitia in the regenerated vessel. This prosthetic vascular graft helped the cell-based graft sustain the pressure of the blood flow but, at the same time, likely limited connective tissue coverage on the surface of the regenerated vessel. This was confirmed by a complete vasculature development after the ePTFE graft was surgically removed after the formation of sustainable vessel from the implanted vascular graft. Therefore, slowly biodegradable materials such as PCL should be applied and studied as part of the hybrid graft for future clinical translation.

In summary, for potential clinical translation we reported a novel on-demand strategy of fabricating TEBV. The reported strategy decouples the integration between TEBV scaffold and the seeded or recruited cells, simplified material selection of scaffolds, and remarkably shorten the time needed of fabricating TEBVs. By applying the strategy, we constructed ADSC vascular grafts that showed normal functionality similar to native blood vessel for the longest observation of more than 1440 days. This cell-based vascular graft shows great promises for clinical trials of human patients.

### Supplementary Information

Below is the link to the electronic supplementary material.

Supplementary file1 (DOCX 1216 kb)

Supplementary file2 (ZIP 44596 kb)

## Data Availability

The datasets generated during and/or analysed during the current study are available from the corresponding author on reasonable request.
