# Supplementary material for: Implantation of Adipose-Derived Mesenchymal Stromal Cells (ADSCs)-Lining Prosthetic Graft Promotes Vascular Regeneration in Monkeys and Pigs
Source: Tissue Eng Regen Med. 2024 Jan 8;21(4):641–51. doi: 10.1007/s13770-023-00615-z (PMC11087433; doi:10.1007/s13770-023-00615-z)
Supplement: Supplementary file 1 — Supplementary file1 (DOCX 1216 kb) [file 13770_2023_615_MOESM1_ESM.docx]

**Supplementary materials and methods**

**Evaluation of mechanical properties of vascular grafts**

The mechanical strength of blood vessels was tested according to ISO7198: 2016(E) Cardiovascular implants and extracorporeal system- Vascular prostheses- Tubular vascular grafts and vascular Patches; Double column bench material test system (Instron 5967, Instron, US) was used to test the longitudinal tensile strength, circumferential tensile strength, probe burst strength, suture strength and strength after repeated puncture. Data were obtained from three separate experiments and expressed as the means±standard errors of the means (SEM). A single factor design was applied to this study. After a significant interaction was detected by analysis of variance (SPSS), the significance of the main effects was further determined by T test. The level of significance was considered when P < 0.05.

**Supplementary Figure 1**

**
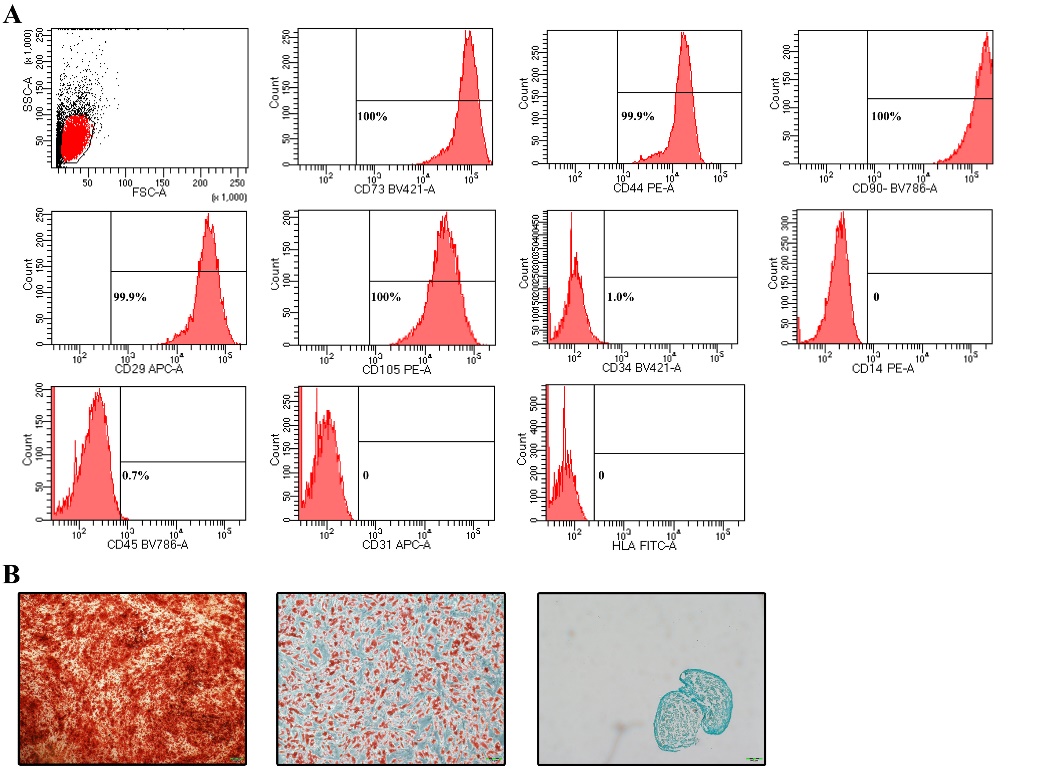
**

**Figure S1.** ADSCs maintained stemness during the culturing process. **A.** The detected markers reflect the stemness of ADSCs, including the positive markers CD90-PE, CD73-PerCP-CyTM5.5, CD29-APC, CD44-PE, and CD105-PE-Cy7 and the negative markers CD45/CD31-FITC and HLA-DR-PerCP-CyTM5.5. **B.** The differentiation of ADSCs cultured in induction media. The results showed that ADSCs differentiated normally into osteoblasts, adipoblasts and chondroblasts.

**Supplementary Figure 2**

**
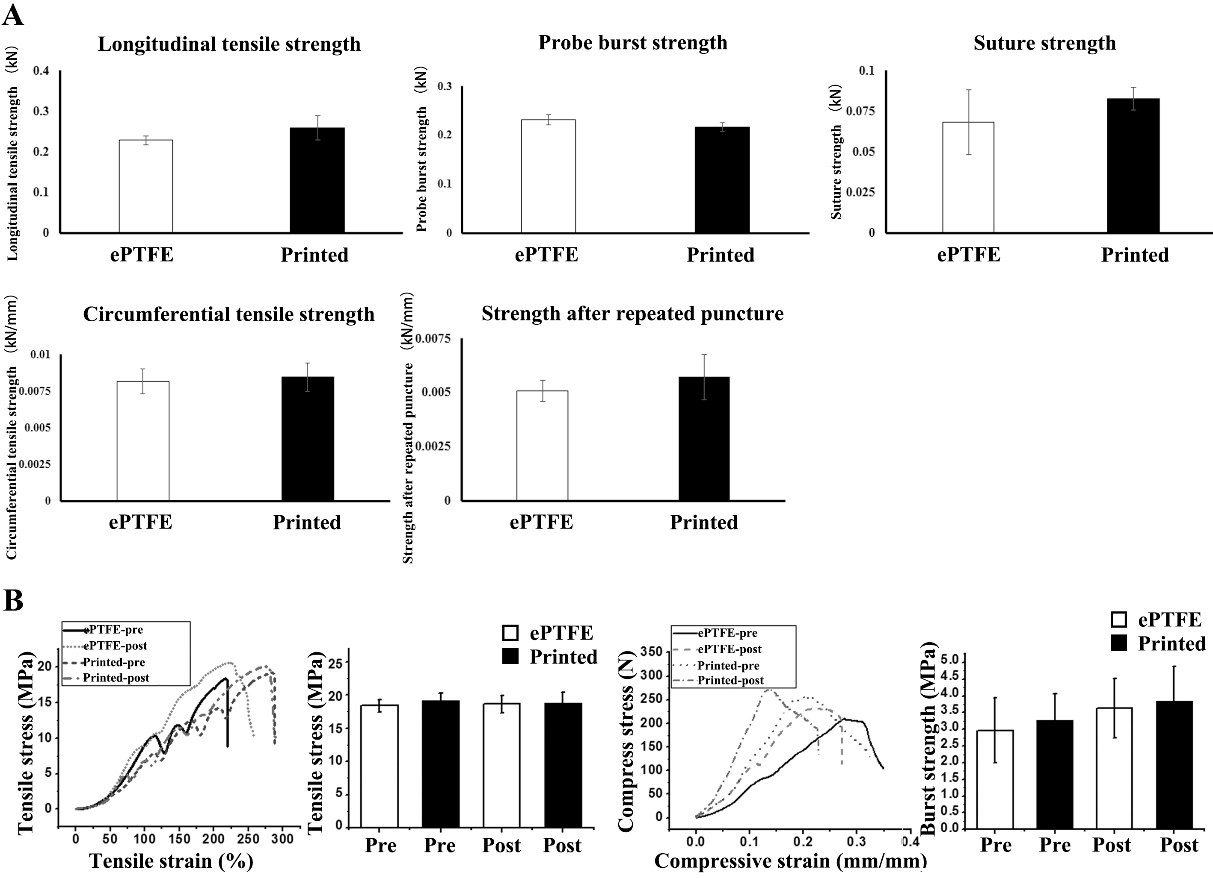
**

**Figure S2.** Detection of the mechanical properties of 3D bioprinted ADSC vascular grafts. **A.** 3D bioprinted ADSC vascular grafts have the same mechanical properties as the ePTFE graft, including longitudinal tensile strength, probe burst strength, suture strength, circumferential tensile strength and strength after repeated puncture. **B.** 3D bioprinted ADSC vascular grafts have the same tensile strain strength and compressive stress as ePTEF grafts before or after implantation. ePTFE: ePTFE prosthetic graft; Printed: 3D bioprinted ADSC vascular grafts; Pre: before implantation; Post: after implantation.

**Supplementary Figure 3**


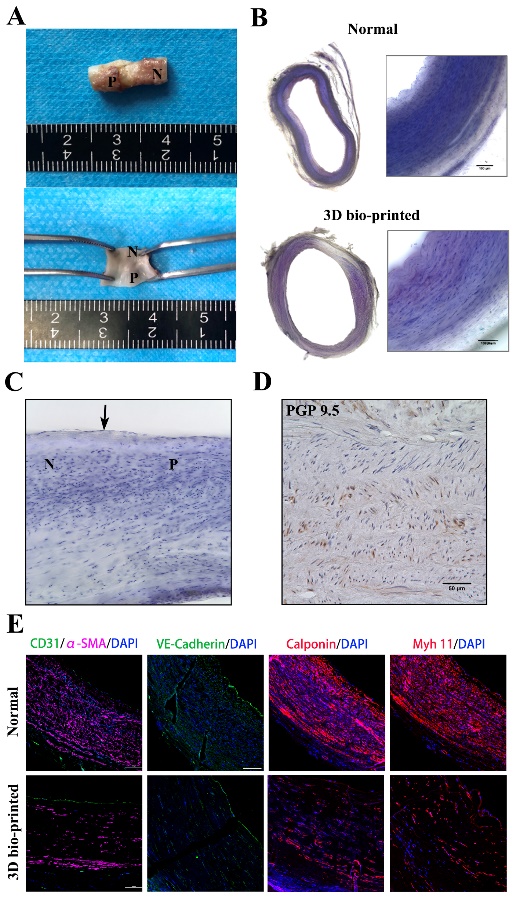


**Figure S3.** Histological, immunofluorescence and immunohistological analyses of 3D bioprinted stem cell vascular grafts implanted in rhesus monkeys for 1420 days. **A.** Representative images of the overall structure of blood vessels. N, normal abdominal aorta; P, 3D bioprinted ADSC vascular graft. **B.** Representative images of cross sections of normal abdominal aorta and the differentiated blood vessel. **C.** Representative images of longitudinal sections of the differentiated blood vessel. Cross sections were stained with hematoxylin for overall structural observation. **D.** The image of immunohistological analyses of bioprinted stem cell vascular grafts. PGP 9.5 was used to label nerve cells. **E.** Representative images of immunofluorescence of the bioprinted stem cell vascular graft by CD31 and VE-cadherin for the identification of endothelial cells and by α-smooth muscle actin (α-SMA), calponin, and Myh to define smooth muscle cells.

**Supplementary Figure 4**


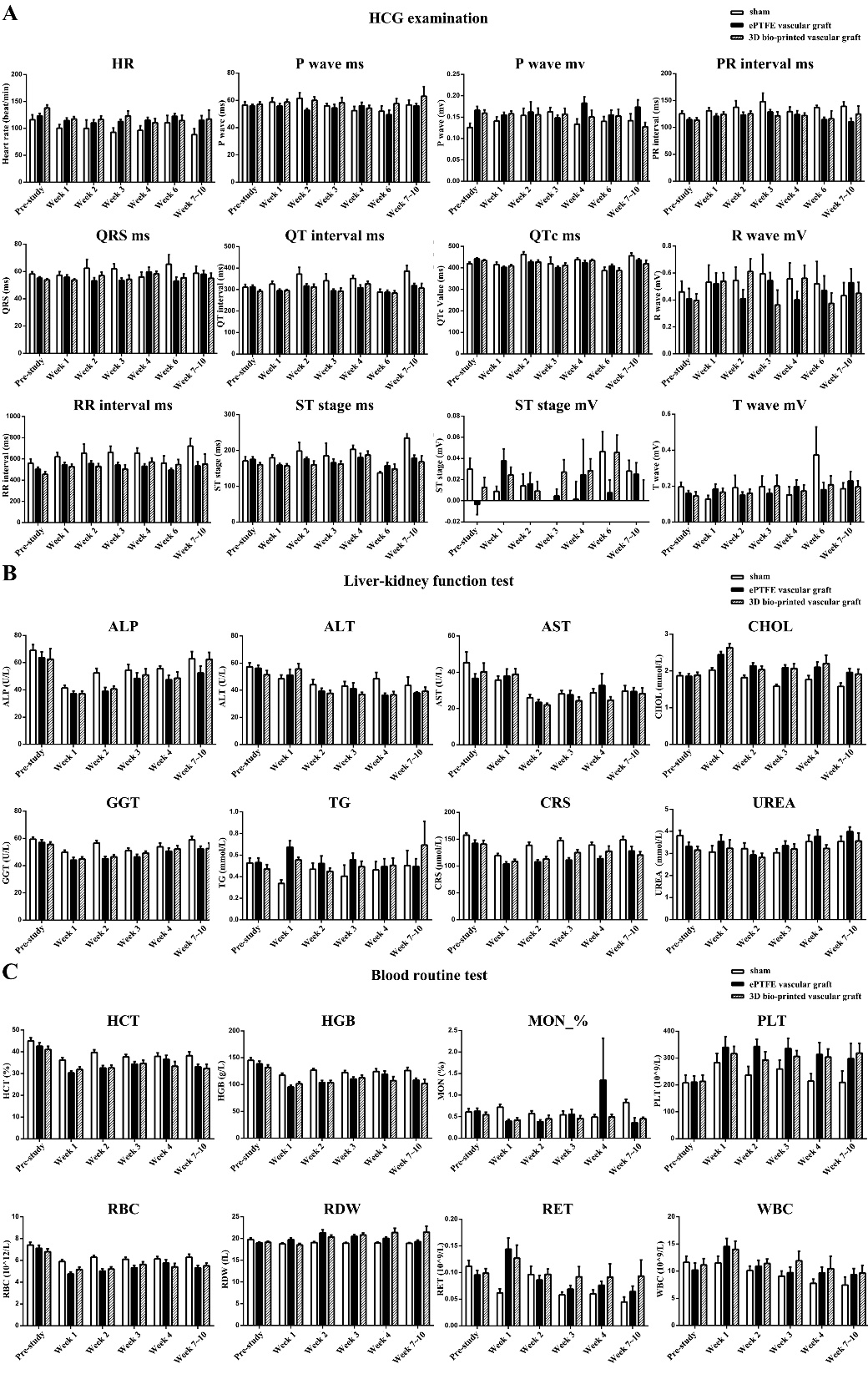


**Figure S4.** The evaluation of physiological function of miniature pigs after hybrid vascular graft implantation. **A.** ECG examination. ECG was performed on Days 0 prior to graft implantation and at weeks 1, 2 and 4 after graft implantation and then once monthly. Animals for ECG evaluation were selected in the same manner as the animals for CTA and ultrasound examination, and the examination was recorded as raw data and within the study file. There were no notable changes in ECG parameters (P wave, R wave, T wave, P-R interval, QRS, Q-T segment, S-T segment, Q-Tc, R-R interval and heart rate) related to the test article (3D bioprinted vascular graft). **B.** Liver-kidney function test. There were no notable changes in liver-kidney function (ALP, ALT, AST, CHOL, GGF, TG, CRS, UREA) related to the test article (3D bioprinted vascular graft). **C.** Routine blood test. There were no notable changes in routine blood tests (HCT, HGB, MON_%, PLT, RBC, RDW, RET, WBC) related to the test article (3D bioprinted vascular graft).
